# BRAF-V600E-Testung beim metastasierten kolorektalen Karzinom und neue, chemotherapiefreie Therapieoptionen

**DOI:** 10.1007/s00292-021-00942-9

**Published:** 2021-05-06

**Authors:** Michael Hummel, Susanna Hegewisch-Becker, Jens Neumann, Arndt Vogel

**Affiliations:** 1 Institut für Pathologie, Campus Charité Mitte, Charité – Universitätsmedizin Berlin, Virchowweg 16/17a, 10117 Berlin, Deutschland; 2 Onkologische Schwerpunktpraxis, Facharztzentrum Eppendorf, Hamburg, Deutschland; 3 Pathologisches Institut, Medizinische Fakultät, Ludwig-Maximilians-Universität München, München, Deutschland; 4 Klinik für Gastroenterologie, Hepatologie und Endokrinologie, Medizinische Hochschule Hannover, Hannover, Deutschland

**Keywords:** Cetuximab, Encorafenib, EGFR-Inhibitoren, Proteinkinaseinhibitoren, Protoonkogene B‑Raf-Proteine, Cetuximab, Encorafenib, BRAF-inhibitors, Protein kinase inhibitors, Proto-oncogene proteins B‑raf

## Abstract

Die Therapie des metastasierten kolorektalen Karzinoms (mKRK) hat in den letzten 25 Jahren tief greifende Veränderungen erfahren. Auf die Zulassung neuerer Chemotherapeutika folgten ab 2005 die ersten zielgerichteten Therapien, die sich gegen den epidermalen Wachstumsfaktorrezeptor (EGFR) bzw. gegen Rezeptoren vaskulärer endothelialer Wachstumsfaktoren (VEGFR) richteten. Mit der fortschreitenden molekularen Charakterisierung des mKRK in den letzten 10 Jahren und der Einteilung der Erkrankung in 4 Konsensus-Subtypen zeichnet sich weiterer Wandel ab, unter anderem durch Einführung speziell entwickelter Proteinkinaseinhibitoren wie auch Immuncheckpoint-Inhibitoren in den Therapiealgorithmus.

Eine angepasste molekularpathologische Testung ist heute für eine leitliniengerechte Behandlung von mKRK-Patienten unabdingbar. Neben der *RAS*-Testung als Voraussetzung für die Therapieentscheidung bezüglich Cetuximab und Panitumumab ist die *BRAF*-Testung äußerst relevant, um – im Falle des Nachweises einer *BRAF-V600E*-Mutation – eine Therapieentscheidung zugunsten der neu zugelassenen, chemotherapiefreien Kombination aus dem BRAF-Inhibitor Encorafenib und Cetuximab treffen zu können. Eine erweiterte Diagnostik sollte auch die Genominstabilität (Mikrosatelliten-Instabilität) einbeziehen. Insgesamt müssen immer mehr molekulare Alterationen simultan untersucht werden, sodass sich zunehmend die Verwendung des fokussierten Next Generation Sequencing empfiehlt.

Diese Übersichtsarbeit beschreibt die prognostische Relevanz der *BRAF*-Testung im Rahmen der molekularpathologischen Diagnostik des mKRK, stellt neue Therapieoptionen zur Behandlung *BRAF*-mutierter mKRK-Patienten vor und erläutert, welche modernen DNA-analytischen und immunohistochemischen Verfahren zur *BRAF*-Diagnostik von mKRK-Patienten zur Verfügung stehen.

## Abstract (en)

In the past 25 years, treatment of metastatic colorectal cancer (mCRC) has undergone profound changes. The approval of newer chemotherapeutics such as irinotecan and oxaliplatin was followed in 2005 by the first targeted therapies, for example, monoclonal antibodies directed against the epidermal growth factor receptor (EGFR), as cetuximab and panitumumab, or the angiogenesis inhibitors bevacizumab, ramucirumab, and aflibercept. With the rapidly progressing molecular characterization of mCRC in the last 10 years and the classification of the disease in four consensus subtypes, further changes are emerging, which will promote, among other things, the introduction of protein-kinase inhibitors developed for specific molecular aberrations as well as immune checkpoint inhibitors into the treatment algorithm.

Thorough molecular pathologic testing is indispensable today for guideline-compliant treatment of mCRC patients. In addition to RAS testing as a precondition for the therapy decision with regard to cetuximab and panitumumab, *BRAF *testing is of considerable relevance to allow decision making with regard to the newly approved chemotherapy-free combination of the BRAF inhibitor encorafenib and cetuximab in cases where a *BRAF-V600E* mutation is detected. Additional diagnostic tests should also include genome instability (microsatellite instability). Overall, more and more molecular alterations need to be investigated simultaneously, so that the use of focused next-generation sequencing is increasingly recommended.

This overview describes the prognostic relevance of *BRAF *testing in the context of molecular pathologic diagnostics of mCRC, presents new treatment options for *BRAF*-mutated mCRC patients, and explains which modern DNA analytical and immunohistochemical methods are available to detect *BRAF *mutations in mCRC patients.

Bei Patienten mit einem kolorektalen Karzinom (KRK) ist die *BRAF*-Testung neben der *RAS*-Testung fester Bestandteil der leitliniengerechten molekularbiologischen Diagnostik vor Einleitung der Erstlinientherapie. Ziel dieses Artikels ist es, eine aktuelle Übersicht zur Bedeutung von *BRAF* als prognostischer und prädiktiver Biomarker zu geben, neue Therapieoptionen für KRK-Patienten mit *BRAF*-Mutationen aufzuzeigen sowie die derzeitig einsetzbaren Diagnoseverfahren zur *BRAF*-Testung darzustellen.

Trotz signifikanter Therapiefortschritte gehört das KRK in Europa weiterhin zu den prognostisch ungünstigsten Krebserkrankungen mit jährlich annähernd 250.000 Todesfällen, die Inzidenz liegt bei über 500.000 Neuerkrankungen [1]. Mit fortschreitender molekularpathologischer Charakterisierung und voranschreitender Klassifizierung des KRK in molekulare Subtypen [2] ergeben sich zunehmend mehr Möglichkeiten zum Einsatz zielgerichteter Therapien, wodurch der molekularen Diagnostik ein immer größerer Stellenwert zukommt.

*BRAF*-Mutationen kommen bei etwa 8–12 % der Patienten mit einem metastasierten KRK (mKRK) vor [3, 4]. Bei über 95 % aller *BRAF*-Mutationen handelt es sich um *BRAF*-V600-Mutationen, bei der in Codon 600 (in Exon 15) des *BRAF*-Gens zumeist eine Substitution von Valin durch Glutaminsäure an Position 1799 erfolgt. Neben dieser häufigsten Mutation, *BRAF*^V600E^, treten auch seltenere Mutationen in Codon 600 auf, bei der ein Austausch des Valins an Position 600 durch Lysin (*BRAF*^V600K^), Asparaginsäure (*BRAF*^V600D^), Methionin (*BRAF*^V600M^) oder Arginin (*BRAF*^V600R^) erfolgt [5]. Klinisch fällt beim Vergleich der *BRAF*-V600-Mutation mit den deutlich selteneren *BRAF*-Mutationen in den Codons 594 und 596 auf, dass erstere häufiger in rechtsseitigen und muzinösen Primärtumoren mit peritonealer Metastasierung gefunden werden. BRAF^594/596^-Tumoren weisen zudem eine günstigere Prognose auf [3]. Die Ausführungen in den nachfolgenden Kapiteln dieser Arbeit beziehen sich immer auf *BRAF*^*V600E*^-Mutationen beim mKRK, soweit nicht explizit anders beschrieben.

B‑Raf ist eine Schlüsselkinase im (Ras/RAF/MEK-)*MAPK*-Signalweg, die in die Regulation des Zellwachstums involviert ist. Die mutationsbedingte Veränderung des *BRAF*-Gens führt zur konstitutiven Aktivierung dieser Proteinkinase, wodurch unkontrollierte Zellteilung auftritt in deren Folge (Neo‑)Angiogenese und Metastasierung zunehmen [6]. Untersuchungen des KRK-Transkriptoms haben zu einer Einteilung des mKRK in 4 Konsensussubtypen („consensus molecular subtypes“, CMS) geführt. *KRAS*-Mutationen treten überwiegend im epithelialen, „metabolischen“ Subtyp CMS3 auf, der sich durch metabolische Dysregulation auszeichnet und auch teilweise chromosomale Instabilität wie auch Mikrosatelliteninstabilität (MSI-H) aufweist [2, 7]. *BRAF*-Mutationen hingegen finden sich häufig im MSI-H/Immun-Subtyp CMS1.

*BRAF*-Mutationen treten nur äußerst selten zusammen mit einer Mutation des *RAS*-Gens auf, die molekulare Testung auf das Vorliegen dieser Mutationen soll laut aktueller S3-Leitlinie möglichst noch vor Einleitung der Erstlinientherapie erfolgen. Dabei kann die *BRAF*-Testung am besten gleichzeitig mit dem *RAS*-Test oder ggf. auch sequenziell nach Ausschluss der *RAS*-Mutation erfolgen [3].

## Onkogene Eigenschaften der *BRAF*-Mutation

*BRAF* ist ein onkogener Treiber bei mKRK-Patienten, der beim malignen Melanom schon lange als therapeutische Zielstruktur etabliert ist. [8]. Die mit einer *BRAF*-Mutation assoziierten serratierten Adenome des Darms weisen molekulare, morphologische, klinische und epidemiologische Charakteristika auf, die sich von denen der Adenome unterscheiden und die sich im Zuge einer „klassischen Adenom-Karzinom-Sequenz“ basierend auf Mutationen des *APC*-Gens entwickeln [9, 10]. Bei der *BRAF*-getriebenen Ausbildung von „sessilen serratierten Adenomen“ (SSA) kommt es zu Störungen der Apoptose der Kryptenepithelien gefolgt von einer Seneszenz mit epigenetischen Promotor(CpG)-Methylierungen und konsekutiv verminderter Expression verschiedener Gene (zum Beispiel h*MLH1, MGMT, p16*) [3, 9]. Bei SSA als Tumorvorstufe und Vorläuferläsionen handelt es sich um flache, kaum aus dem Schleimhautniveau aufragende Polypen, die zumeist im rechtsseitigen Kolon auftreten und auch endoskopisch nur schwer zu erkennen sind [3, 9]. Patienten mit großen serratierten Adenomen weisen ein erhöhtes Risiko der kolorektalen Karzinomentstehung auf. Bei Frauen mit SSA ist das Risiko gegenüber Männern um den Faktor 5 erhöht [3].

### Klar negativer prognostischer Faktor beim KRK

Beim mKRK gilt erhöhtes Alter ebenso als negativ prognostisches Kennzeichen wie eine Lage des Tumors proximal der linken Flexur [11]. In einer großen retrospektiven Fallserie wurde der Einfluss von *BRAF*-Mutationen und MSI‑H auf die metastatische Ausbreitung und Prognose von mKRK untersucht: *BRAF*-mutante Tumoren und hier wiederum die V600E-Mutation sind gegenüber dem *BRAF*-Wildtyp mit einer signifikant schlechteren Überlebensprognose assoziiert (Median 10,4 *versus* 34,7 Monate; Hazard Ratio [HR] = 10,66, *p* < 0,001) wie auch mit einer höheren Rate an peritonealen Metastasen und distanten Lymphknotenmetastasen [12]. Die prognostisch äußerst negative Auswirkung von *BRAF*^*V600*^ wurde auch bei randomisierten kontrollierten Studien übereinstimmend berichtet [4, 13, 14]. Eine ausführliche Diskussion des prognostischen Einflusses von *BRAF*-Mutationen in Abhängigkeit von der Mikrosatellitenstabilität bzw. -instabilität als weiterem Biomarker findet sich in 2 aktuellen Übersichtsarbeiten [6, 15]. MSI‑H tritt, mit Ausnahme des hereditären nicht-Polyposis-assoziierten kolorektalen Karzinoms (HNPCC), mit einer geschätzten Häufigkeit von nur 4–8 % bei mKRK-Patienten auf [4]. Bei gleichzeitigem Auftreten von *BRAF*-Mutationen und MSI‑H – die Häufigkeit liegt reziprok jeweils bei ungefähr einem Drittel – handelt es sich um sporadische Defekte der DNA-Mismatch-Reparatur (dMMR) [3, 4]. MSI-H-Patienten haben insgesamt eine bessere Prognose als Patienten mit Mikrosatellitenstabilität (MSS)[16]. Trotz insgesamt kleiner Fallzahlen deutet die derzeitig verfügbare klinische Evidenz darauf hin, dass mKRK-Patienten mit einer *BRAF*^*V600E*^-Mutation und MSI‑H eine insgesamt bessere Prognose haben als Patienten mit einer *BRAF*^*V600E*^-Mutation und MSS-Befundung, wobei in der metastasierten Situation die Kombination BRAF^V600E^ und MSS zu überwiegen und somit die schlechte Prognose der BRAF-Mutation führend zu sein scheint [6, 13, 17–20].

### Prädiktiver Wert bezüglich bisheriger konventioneller Therapien unklar

Vor dem Hintergrund eines sich mutuell nahezu ausschließenden Auftretens von *BRAF*- und *RAS*-Mutationen [21] und der nachgewiesen negativen prädiktiven Bedeutung von *RAS*-Mutationen für den Einsatz von Anti-EGFR-Therapien stellt sich auch für *BRAF*-Mutationen die Frage der prädiktiven Relevanz bezüglich des Einsatzes der beiden monoklonalen Antikörper Cetuximab und Panitumumab. Beide sind beim KRK ausschließlich für den Einsatz bei Patienten mit *RAS*-Wildtyp zugelassen. Für den Einsatz von Anti-EGFR-Therapien bei Patienten mit einer *BRAF*-Mutation liegen nur begrenzte Daten aus Subgruppenanalysen größerer konfirmatorischer Studien (Tab. 1) sowie rein retrospektive, aus klinischen Praxisdaten erhobene Fallserien vor [22, 23].

**Table 1:** 

Studie/Phase (bzw. Typ) Studie	Vergleich	„Backbone“ (Therapie)	N_ITT, total_ [N_BRAF-mut._] (soweit ermittelt)^a^	BRAF-Assessment^b^ (diagnost. Methode)	OS (Monate)	PFS (Monate)	ORR (%)	HR [95%KI]	Referenz
**Anti-EGFR-Therapien**									
*Erstlinientherapie*									
Crystal + OPUS/III (R-SGA)	Cetuximab + CTx *vs*. CTx	FOLFIRI (Crystal), FOLFOX4 (OPUS)	1535 [32 *vs.* 38]	PNA-clamping PC	14,1 *vs*. 9,9	7,1 *vs*. 3,7	22 *vs*. 13	OS: 0,62 [0,36–1,06] PFS: 0,67 [0,34–1,29]	Bokemeyer et al. [36]
PRIME/III (R-SGA)	Panitumumab + CTx vs. CTx	FOLFOX	1183 [24 *vs.* 29]	PNA-clamping PC	10,5 *vs*. 9,2	6,1 *vs*. 5,4	NA	OS: 0,90 [0,46–1,76] PFS: 0,58 [0,29–1,15]	Douillard et al. [37]
FIRE-3/III (R-SGA)	Cetuximab + CTx vs. Bevacizumab + CTx	FOLFIRI	752 [23 *vs.* 25]	Pyrosequencing	12,3 *vs*. 13,7	6,6 *vs*. 6,6	52 *vs*. 40	OS: 0,79 [0,43–1,46] PFS: 0,84 [0,47–1,51]	Stintzing et al. [38]
*Zweitlinientherapie*									
20020181/III (R-SGA)	Panitumumab + CTx vs. CTx	FOLFIRI	1186 [22 *vs.* 23]	PCR/Sanger	5,7 *vs*. 4,7	2,5 *vs*. 1,8	NA	NA	Peeters et al. [39]
PICCOLO/III (R-SGA)	Panitumumab + CTx vs. BSC	Irinotecan	460 [37 *vs.* 31]	PCR/Pyrosequencing	NA	NA	11 *vs*. 6	NA	Seymour et al. [40]
*Therapierefraktäre Patienten (≥* *2 Vortherapien)*									
20020408/III (R-SGA)	Panitumumab *vs*. BSC	Ø	463 [18^c^]	PCR (Sequenzierung)	NA	NA	0 (*vs*. 0)	NA PFS: 0,34 [0,09–1,24]	Peeters et al. [41]
CO.17/III (R-SGA)	Cetuximab *vs*. BSC	Ø	572 [4 *vs.* 6]	PCR (Sequenzierung)	1,8 *vs*. 3,0	NA	0 *vs*. 0	OS: 0,84 [NA–NA] PFS: 0,76 [NA–NA]	Karapetis et al. [42]
**Anti-VEGF-Therapien**									
*Erstlinientherapie*									
TRIBE/III (R-SGA)	Bevacizumab; Vgl. zweier CTx-Backbones	FOLFOXIRI vs. FOLFIRI	508 [16 *vs.* 12]	Pyrosequencing	19,0 *vs*. 10,7	7,5 *vs*. 5,5	56 *vs*. 42	OS: 0,54 [0,24–1,20] PFS: 0,57 [0,27–1,23]	Cremolini et al. [28]
Loupakis et al./II	Bevacizumab + CTx	FOLFOXIRI	25 [25^d^]	HRM-Analyse/Sequenzierung	24,1	9,2	60	NA	Loupakis et al. [27]

*BSC* „best supportive care“, *CTx* Chemotherapie, *EGFR* „epidermal growth factor receptor“, *HR* Hazard Ratio, *HRM* „high resolution melting“, *NA* nicht angegeben, *N*_ITT_ Gesamtzahl der in der Studie randomisierten Patienten, *ORR* Gesamtansprechrate, *OS* Gesamtüberleben* , PCR* Polymerase-Kettenreaktion, *PFS* progressionsfreies Überleben, *R‑SGA* retrospektive Subgruppenanalyse, *vs*. versus, *VEGF* „vascular endothelial growth factor“

^a^Prozentsatz *BRAF*-mutierter Patienten bezieht sich auf die Gesamtzahl der Patienten, für die Ergebnisse/Gewebe zu einer *BRAF*^*V600E*^-Mutationsanalyse vorlagen (*BRAF*-mutiert *versus BRAF*-Wildtyp)

^b^Angaben gemäß Pietrantonio et al. [24]

^c^Anzahl Patienten im experimentellen Studienarm (d. h. Panitumumab-Arm) mit bekannter *BRAF*^V600E^-Mutation

^d^Validierungskohorte (*N* = 25), hierin 15 prospektiv in diese Studie eingeschlossenen Patienten und 10 Patienten aus einer vorherigen Studie, bei denen der BRAF-Status retrospektiv bestimmt wurde

Einen klinischen Benefit haben 2 sich in Teilen überlappende Metaanalysen dieser Daten der Anti-EGFR-Antikörpertherapie bei *RAS*-plus *BRAF*-Wildtyp-Patienten bestätigen können. Bei *RAS*-Wildtyp Patienten mit einer *BRAF*-Mutation zeigten sich in der Gesamtheit der Daten allerdings nur bedingte, nicht signifikante klinische Vorteile beim progressionsfreien Überleben wie auch beim Gesamtüberleben [24, 25]. Die derzeitige Datenlage wird auch in dem Sinne kontrovers diskutiert, dass diese Daten eine Exklusion von Anti-EGFR-Antikörpern für die Patientengruppe mit *BRAF*^*V600E*^-Mutation gleichfalls nicht rechtfertigen. Die Daten aus der deutschen, randomisierten Phase-II-Studie VOLFI zum Vergleich von Panitumumab plus Chemotherapie *versus* alleiniger Chemotherapie in der Erstlinie zeigten, dass die Hinzugabe von Panitumumab zur Chemotherapie die Ansprechrate (ORR) bei den 14 Patienten mit *BRAF*^*V600E*^-mutierten Tumoren tendenziell erhöhte (Odds Ratio 14,93; 95 %-KI 1,03–200,00) [26].

Für die im klinischen Alltag des mKRK bedeutsame und unabhängig vom *RAS*-Status einsetzbare Anti-VEGF-Therapie – wie die Anti-EGFR-Therapie in Kombination mit (in der Erstlinie zumeist) Oxaliplatin-haltiger beziehungsweise (in der Zweitlinie zumeist) Irinotecan-haltiger Chemotherapie gegeben – stehen bisher keine bzw. nur indirekte Vergleiche zur Verfügung, sodass der prädiktive Wert einer *BRAF*-Testung hier unklar ist. Die Ergebnisse einer kleinen Phase-II [27] sowie Subgruppenanalysen zweier großer Phase-III-Studien [28, 29] lassen in der Gesamtschau keine eindeutige Beurteilung der Bedeutung einer intensivierten Chemotherapie (FOLFOXIRI) bei *BRAF*-mutierten Patienten zu. Eine Metaanalyse von 5 randomisierten Studien mit heterogenen Fallzahlen (*n* = 70–679) und unterschiedlichen Fragestellungen kam jüngst zu dem Ergebnis, dass bei *BRAF*-mutierten Patienten – bei weiterhin insgesamt kleinen Fallzahlen – in der Erstlinie eher kein gesteigerter Nutzen durch die intensivierte Kombinationstherapie besteht [30]. Eine im Herbst 2020 vorgestellte Metaanalyse der ARCAD-Studiengruppe mit gepoolten Daten zweier Studien, die den Ansatz Chemotherapie plus Anti-EGFR- bzw. plus Anti-VEGF-Therapie in der Erstlinientherapie des mKRK direkt verglichen, konnte für die Subgruppe der *BRAF*-mutierten Patienten (*n* = 138) keinen relevanten Unterschied im Gesamtüberleben aufzeigen zwischen Bevacizumab-basierter Behandlung und Cetuximab-basierter Therapie (HR = 1,01, 95 %-KI 0,69–1,48) [31]. Der Wert der Anti-VEGF-Therapie mit Bevacizumab an sich wie auch die prädiktive Rolle von BRAF für die Einleitung einer Bevacizumab-basierten Therapie erfordern noch weitergehende Untersuchungen (Tab. 1).

**Figure 1:**
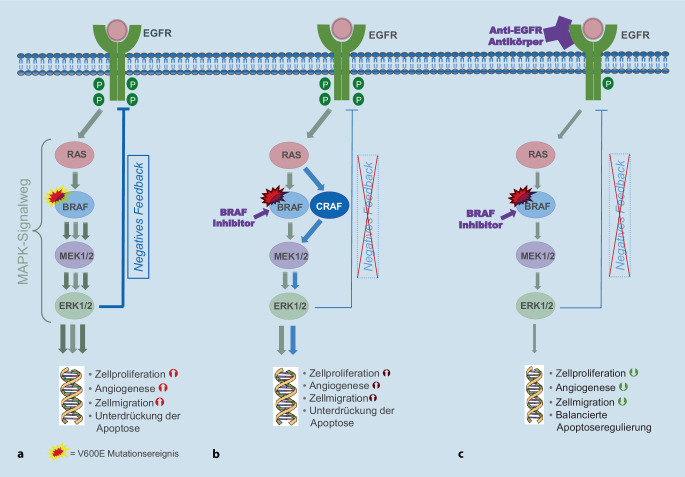

Obwohl zielgerichtete Tyrosinkinaseinhibitoren in der klinischen Routine seit 2011 mit viel Erfolg beim *BRAF*^*V600*^-mutierten Melanom eingesetzt werden, zeigte sich das *BRAF*-mutierte mKRK wenig sensitiv bei monotherapeutischem Einsatz [32, 33]. Die Ursache hierfür scheinen KRK-spezifische Resistenzmechanismen in der MAPK-Signalkaskade zu sein. In-vitro-Studien belegten eine Unterdrückung des zwischen der ERK-Kinase und dem EGFR bestehenden negativen Feedbackloops unter BRAF-Monotherapie bei insgesamt hoher EGFR-Expression und möglicherweise stärkerer Aktivierung des Rezeptors durch seine Liganden (Abb. 1; [33, 34]). Dies resultiert in einer Reaktivierung des EGFR-Signalwegs, z. B. unter Umgehung des mutierten *BRAF*-Proteins über CRAF. Die zusätzliche Inhibierung des EGFR-Signalwegs durch gleichzeitige Gabe einer gegen EGFR-gerichteten Therapie zusätzlich zur *BRAF*-Blockade erscheint daher wichtig, um die mehrgleisigen Resistenzmechanismen innerhalb des MAPK-Signalwegs zu blockieren [33, 35].

## Therapieoptionen beim *BRAF*-mutierten mKRK

Die Therapie des mKRK ist bisher in der Erst- wie Zweitlinie generell durch Verwendung von Kombinationschemotherapien geprägt und, wie im Kapitel zuvor dargestellt, zumeist – bei linksseitigen *RAS*-Wildtyp-Tumoren – in Verbindung mit einer EGFR-Antikörpertherapie [3, 4]^1^. Die gültigen Therapieempfehlungen beim mKRK räumen dem Allgemeinzustand der häufig älteren Patienten hinsichtlich der Therapieentscheidung großen Raum ein und unterscheiden – bei den Patienten, die fit genug für eine systemische Therapie sind – zwischen den Behandlungszielen „Zytoreduktion“, mit dem Ziel einer Reduktion der Tumormasse, sowie „Krankheitskontrolle“ mit dem Ziel des Herauszögerns weiterer Progression.

### Erstlinientherapie beim BRAF-mutierten mKRK

Die Kombination eines Antimetaboliten (5-Fluorouracil plus Leucovorin als Folinsäurederivat) mit einer die DNA-Replikation störenden Platinverbindung (Oxaliplatin) sowie einem Topoisomerase-I-Inhibitor (Irinotecan) und dem Angiogenesehemmer (anti-VEGF) Bevacizumab in *BRAF*-mutierten mKRK-Patienten mit gutem Allgemeinzustand ist derzeit europäischer Leitlinienstandard für diese Subentität [4]. Hinsichtlich der Evidenzniveaus in der *BRAF*-mutierten Subgruppe ist allerdings zu beachten, dass die Leitlinienempfehlung nur auf einer sehr geringen Patientenzahl (*N* = 28) aus der TRIBE-Studie beruht und somit mit entsprechenden Unsicherheiten behaftet ist [28]. In der *BRAF*-Subgruppe dieser Phase-III-Studie betrug das Gesamtüberleben unter FOLFOXIRI plus Bevacizumab 19 Monate, 56 % der Patienten sprachen auf die Therapie an. Jedoch wurde kein signifikanter Unterschied gegenüber der Vergleichstherapie aus FOLFIRI plus Bevacizumab erreicht (Tab. 1; [28]). Dieser Erstlinienstandard ist momentan Gegenstand kontroverser Diskussion. Eine kürzlich publizierte Metaanalyse aus 5 randomisierten Studien zu FOLFOXIRI plus Bevacizumab *vs*. Doublet-Chemotherapie plus Bevacizumab ergab bei *BRAF*-mutierten Tumoren einen nicht signifikanten Trend zugunsten des weniger intensiven Regimes (*n* = 115; HR: 1,12, 95 %-KI: 0, 75–1,68) [30].

Aufgrund der schlechten Prognose von mKRK-Patienten mit einer *BRAF*^*V600E*^-Mutation kommt dem Konzept, die Erstlinienbehandlung beim mKRK möglichst aggressiv unter Verwendung fast des gesamten Arsenals an Therapiemodalitäten anzugehen, auf Basis der aktuellen Empfehlungen klinische Bedeutung zu – insbesondere, wenn eine Zytoreduktion angestrebt wird. Allerdings ist unklar, in welchem Umfang in Deutschland Patienten diese intensivierte und mit entsprechenden Nebenwirkungen behaftete Erstlinientherapie erhalten.

Wie zuvor geschildert ist darüber hinaus die Frage des Einsatzes der chemotherapiekombinierten Anti-EGFR-Therapie bei *BRAF*-mutierten Tumoren auf Basis der nicht eindeutigen Ergebnisse zweier Metaanalysen ebenfalls Gegenstand einer kontroversen Debatte [24, 25].

### Neue chemotherapiefreie, zielgerichtete Optionen in der Zweit- bzw. Drittlinientherapie

Aufgrund der bis dato limitierten Therapieoptionen nach erfolgter Erstlinientherapie konnten sich in den Leitlinien für die Zweit‑/Drittlinientherapie des *BRAF*-mutierten mKRK bislang keine klaren Empfehlungen herausbilden [3, 4]. Die deutsche S3-Leitlinie von 2019 schrieb hierzu: „*individuelle (derzeitig) nicht zugelassene Therapieansätze, z.* *B. mit einem BRAF-inhibitor, MEK-Inhibitor und Anti-EGFR-Antikörper oder wenn möglich die Behandlung im Rahmen einer klinischen Studie *(sind)* in Betracht zu ziehen*“; wobei die hier genannten Therapieoptionen bislang keine Zulassung für diese Indikation besaßen.

Mit der im Juni 2020 erfolgten EU-Zulassung der Kombinationstherapie des *BRAF*-Inhibitors Encorafenib und des Anti-EGFR-Antikörpers Cetuximab steht eine chemotherapiefreie, zielgerichtete Doppelblockade nun für die Routineversorgung zur Verfügung. Die Zulassung der Kombination gilt für Patienten mit einem mKRK mit *BRAF*^*V600E*^-Mutation, die zuvor eine systemische Vortherapie erhalten haben [43].

**Figure 2:**
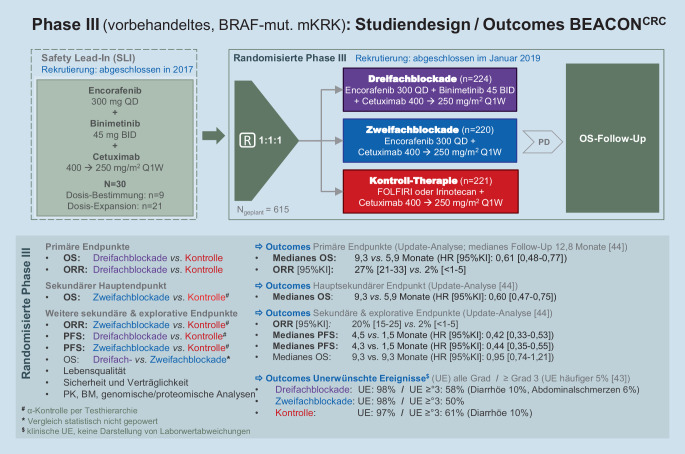

Die der Zulassung zugrunde liegende Phase-III-Studie BEACON CRC untersuchte die Dreifachblockade aus Encorafenib und Cetuximab plus dem MEK-Inhibitor Binimetinib und der Zweifachblockade aus Encorafenib und Cetuximab im Vergleich zu einer Kontrolltherapie aus Irinotecan-basierter Chemotherapie plus Cetuximab in Patienten mit BRAF^V600E^-mutiertem mKRK, die zuvor 1 oder 2 palliative Therapielinien erhalten hatten (Abb. 2; [44]). Primäre Endpunkte waren Tumoransprechen (ORR) und Gesamtüberleben (OS) der Dreifachblockade *vs*. Kontrolltherapie. Die Studie war auf den Endpunkt OS (für Therapiegruppe „Zweifachblockade“ versus Kontrollgruppe) hin gepowert. Weitere sekundäre Endpunkte (OS, ORR und progressionsfreie Überleben [PFS] – jeweils Zweifachblockade vs. Kontrolltherapie sowie das PFS der Dreifachblockade *vs.* Kontrolle) waren ebenfalls alpha-kontrolliert und somit von konfirmatorischer Relevanz. Insgesamt wurden 665 Patienten im Verhältnis 1:1:1 in die 3 Therapiearme randomisiert.

Die primären Endpunkte der Studie wurden erreicht. Die Kombination aus Encorafenib, Cetuximab und Binimetinib zeigte in der primären Analyse (medianes OS-Follow-up 7,8 Monate) eine ORR von 26 % [95 %-KI 18–35] *vs*. 2 % [95 %-KI < 1–7] unter der Kontrolltherapie (*p* < 0,001). Das mediane OS der Dreifachblockade gegenüber der Kontrolle betrug 9,0 *vs*. 5,4 Monate (HR [95 %-KI]: 0,52 [0, 39–0,70]; *p* < 0,001) [44]. Für den alpha-kontrollierten Vergleich von OS der Zweifachblockade *vs*. Kontrolltherapie konnte für Encorafenib plus Cetuximab eine Verlängerung des medianen OS um 3 Monate (8,4 *vs.* 5,4 Monate; HR [95 %-KI]: 0,60 [0, 45–0,79], *p* < 0,001) gezeigt werden; die ORR betrug 20 % (95 %-KI: 13–29) *vs*. 2 % (95 %-KI: < 1–7; *p* < 0,001).

Die Verträglichkeit der Zweifachblockade stellte sich gegenüber der Dreifachblockade sowie gegenüber der Kontrolle etwas günstiger dar (Abb. 2). Das Sicherheitsprofil der Kombination aus Encorafenib und Cetuximab war gut handhabbar und zeigte die erwartbaren klassentypischen Ereignisse. Die am häufigsten beobachteten unerwünschten Ereignisse (UE) waren: erhöhtes Kreatinin (50 %), Übelkeit (34 %), Diarrhoe (33 %), verringertes Hämoglobin (32 %), Fatigue (30 %), akneiforme Dermatitis (29 %) und verminderter Appetit (27 %) [44].

Aufgrund der vergleichbaren Wirksamkeitsergebnisse der Dreifach- gegenüber der Zweifachblockade bei etwas günstiger Verträglichkeit von Encorafenib + Cetuximab sprach die Europäische Arzneimittelbehörde EMA im Juni 2020 die Zulassung für die Zweifachkombination aus [43].

Eine aktuelle Updateanalyse nach einem medianen Follow-up von 12,8 Monaten bestätigte die oben genannten Ergebnisse bei weitgehend unverändertem Verträglichkeitsprofil [45]. Das mediane Gesamtüberleben betrug hierbei 9,3 Monate (95 %-KI: 8,2–10,8) unter Dreifachblockade, 9,3 Monate bei Behandlung mit der nun neu zugelassenen Zweifachblockade aus Encorafenib und Cetuximab (95 %-KI: 8,0–11,3) sowie 5,9 Monate (95 %-KI: 5,1–7,1) in der Kontrollgruppe.

**Figure 3:**
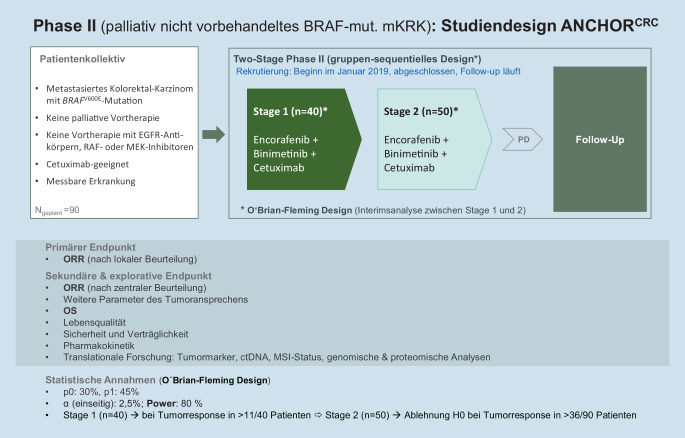

Die zielgerichtete Dreifachblockade aus Encorafenib, Cetuximab und Binimetinib wird derzeit im Rahmen der zweistufigen ANCHOR CRC Phase-II-Studie als Erstlinientherapie weiter untersucht (Abb. 3).

## *BRAF*-Diagnostik

Aus der zuvor beschriebenen klinischen Situation ergibt sich die Notwendigkeit einer Testung auf das Vorliegen von *RAS*- und *BRAF*-Mutationen beim mKRK, um eine an den Mutationsstatus angepasste Therapiesequenzplanung – im Fall *BRAF*^*V600E*^-mutierter Patienten unter Berücksichtigung der neuen Therapieoption Encorafenib plus Cetuximab – vornehmen zu können.

**Figure 4:**
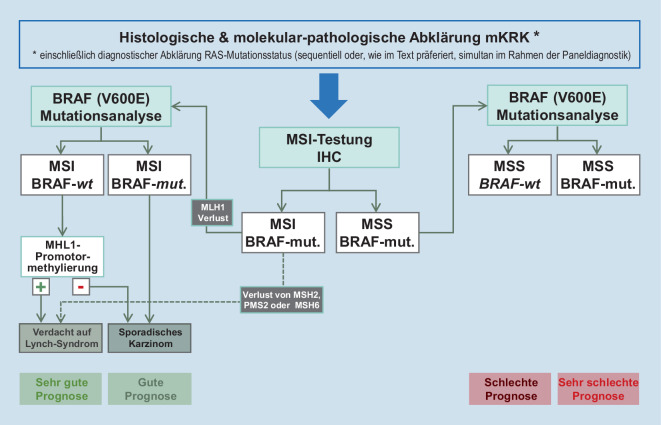

Die Leitlinien empfehlen eine solche Diagnostik vor der Einleitung der Erstlinientherapie des mKRK bzw. bereits bei der Erstdiagnose des KRK, um ggf. zusammen mit einer dMMR-Testung das Vorliegen eines Lynch-Syndroms ausschließen zu können [3, 4]. Solche hereditären KRK ohne Polyposis (HNPCC) stellen wie *BRAF*-mutierte Tumore einen biologisch unterschiedlichen Subtyp des KRK dar. Bei Vorliegen einer *BRAF*-Mutation in einem dMMR/MSI-H-Tumor kann ein Lynch-Syndrom weitestgehend ausgeschlossen werden. Die zeitgerechte Bestimmung des *BRAF*-Mutationsstatus ist somit von diagnostischer und insbesondere auch therapeutischer Relevanz (verzögerungsfreier Start der Erstlinie; Planung der Therapiesequenz) und unterstützt zudem bei der diagnostisch wichtigen Unterscheidung, ob die Defekte der Mismatchreparatur somatischer oder genetischer Natur sind [46–48]. Die Annahme eines sporadischen Tumors und somit der Ausschluss von HNPCC/Lynch-Syndrom kann durch Analyse der *MLH1*-Promotormethylierung unterstützt werden, da das Vorliegen einer solchen Methylierung die Diagnostik eines sporadischen MSI-H-Status zusätzlich absichert (Abb. 4). Die BRAF-Analytik kann entweder zeitgleich zur RAS-Analytik oder stufendiagnostisch nach Ausschluss einer *RAS*-Mutation erfolgen. Heutzutage empfiehlt sich allerdings erstere Vorgehensweise mittels entsprechender Genpaneldiagnostik unter Verwendung von fokussiertem Next Generation Sequencing (NGS).

### Probenvorbereitung

Im Rahmen der klinischen Befundklärung werden Proben zumeist im Rahmen einer Darmspiegelung oder der operativen Entfernung des Primärtumors erhalten. Nach Fixierung in 10 % neutral gepuffertem Formalin (4 % Formaldehyd) für 24–48 h und Einbettung in Paraffin sind die Proben nach Extraktion der DNA für die unter Abb. 4 aufgeführten Untersuchungen sehr gut geeignet. Für die molekulare Bestimmung des MSI-H-Status sollten auch gesunde, tumorferne Gewebeanteile asserviert und untersucht werden [4]. Im Falle von Gewebeproben mit einem geringen prozentualen Tumorzellanteil wird zur Anreicherung der Tumorzellen eine Makrodissektion vor der DNA-Extraktion angeraten.

### Molekularpathologische, DNA-analytische Verfahren

Zur Bestimmung des *BRAF*^*V600E*^-Mutationsstatus stehen unterschiedliche Testmethoden mit verschiedener Spezifität und Sensitivität zur Verfügung. Mit klassischen Testverfahren (Tab. 2) wie der Sanger-Sequenzierung lassen sich 99 % aller Mutationen erkennen mit einer Spezifität von 100 %. Es muss jedoch darauf geachtet werden, dass ein Tumorzellanteil von 20–30 % nicht unterschritten werden darf. Der Nachweis der *BRAF*^*V600E*^-Mutation mittels High-Resolution-Melting(HRM)-Analyse oder Pyrosequenzierung ist sensitiver als die Sanger-Sequenzierung, sodass ein Tumorzellgehalt von etwa 10–15 % (5 % Sensitivität) ausreichend erscheint [49, 50]. Ähnliche Sensitivitäten weisen auch kommerzielle Tests wie ThxID-BRAF-Kit® (bioMérieux, Marcy-l’Étoile, Frankreich;*V600E* sowie *V600K),* cobas 4800® *BRAF* Mutation Test (F. Hoffmann-La Roche AG, Basel, Schweiz; nur V600E) oder Idylla™ BRAF Mutation Test (Biocartis NV, Mechelen, Belgien) auf (Tab. 3; [51–53]).

**Table 2:** 

	Sanger	Pyro-Sequenzierung	HRM	NGS
Typ Diagnostik	Laborbasiert	Laborbasiert	Laborbasiert	Laborbasiert
Zulassung^a^	Nicht erforderlich	Nicht erforderlich	Nicht erforderlich	EU: nein/USA: teils ja
Indikation	Multipel	Multipel	Multipel	Multipel
Selektivität^b^	Ja	Ja (Codon 600)	Ja	Ja
Spezifität	100 %	90 %	100 %	100 %
Sensitivität	92 %	> 98 %	98–100 %	≈ 100 %
Nachweisgrenze	10 %	5 %	6 %	1/5 %
In-Lab-Zeit *(Turnaround)*	2–3 Tage	≈ 2 Tage	≈ 1 Tag	2–4 Tage

*HRM* „high resolution melting“, *NGS* Next Generation Sequencing

^a^Im Sinne von CE-Kennzeichnung (EU) bzw. Premarket Approval (USA)

^b^Im Sinne seltener *BRAF*-Mutationen (Non-*BRAF*^V600E^)

**Table 3:** 

	THxID® BRAF Kit (bioMérieux, Marcy-l’Étoile, Frankreich)	cobas® 4800 BRAF V5600 Mutation Test (F. Hoffmann-La Roche AG, Basel, Schweiz)	Idylla™ BRAF Mutation Test (Biocartis NV, Mechelen, Belgien)	Qiagen *therascreen*® BRAF V600E RGQ PCR kit (Qiagen NV, Venlo, Niederlande)	Foundation One® CDx (Roche Pharma AG, Grenzach-Wyhlen, Deutschland)
Typ Diagnostik	Standardisiert	Standardisiert	Standardisiert	Standardisiert	NGS
Zulassung	USA (CDx), EU (CE)	USA (CDx), EU (CE)	USA (CDx), EU (CE)	USA (CDx), EU (CE)	USA (CCDx)
FDA PMA N^o^ (Jahr)	*P120014* (2012)	*P110020/S016* (2016)	(510(k) Notifizierung nicht erforderlich)	*P190026* (2020)	*P170019* (2017)
Indikation	Melanom	Melanom	Multiple Indikationen	**CRC**	Multiple Mutationen und Indikationen
Selektivität	Nur V600E, V600K	Nur V600E	V600E/E2/D und V600K/R/M	Nur V600E	Nur V600E, V600K
Sensitivität	> 96 % ^V600E^; > 92 % ^V600K^	> 98 %	> 98 %	> 98 %	100 %
Spezifität	100 %	> 98 %	> 98 %	100 %	≈ 100 %
Nachweisgrenze	5 % ^V600E^, 5 % ^V600K^	5–7 % ^V600E^, > 35 % ^V600K^	Nicht spezifiziert	8 %	2 %
In-Lab-Zeit (Turnaround)	1 Tag	1 Tag	2–4 h	1 Tag	≈ 3–5 Tage

*CDx* Companion Diagnostic, *CDDx* Companion und/oder Complementary Diagnostic, *NGS* Next Generation Sequencing, *PMA* Premarket Approval (USA)

In den letzten Jahren haben sich in der molekularen Diagnostik verstärkt NGS-Verfahren durchgesetzt, mit denen sich eine Auswahl von diagnose- und therapierelevanten Genen/Genabschnitten („targeted NGS“) simultan mit hoher Sensitivität und Spezifität nachweisen lassen. Daher wird der Nachweis einer *BRAF*-Mutation beim KRK häufig nicht mehr als isolierter Einzelnachweis durchgeführt, sondern eingebettet in den parallelen Nachweis anderer molekularer Alterationen, wie zum Beispiel *KRAS* und *NRAS*. Die Sensitivität der NGS-basierten Verfahren ist grundsätzlich sehr hoch (etwa 1 %), wird aber durch Artefakte, die im Rahmen der Formalinfixierung entstehen, eingeschränkt. In vielen Laboren wird deshalb ein Schwellenwert von 5 % Varianten-Allelfrequenz (VAF) vorgegeben, der unter bestimmten Bedingungen gegebenenfalls unterschritten werden kann [49, 50]. Als NGS-Plattformen stehen heute primär ThermoFisher Scientific (Waltham, USA) und Illumina (San Diego, USA) zur Verfügung. Mit beiden Plattformen lassen sich zahlreiche kommerzielle oder eigene Genpanel einsetzen, wobei sowohl amplikonbasierte Verfahren (Multiplex-PCR) als auch Hybrid-Capture-Verfahren zur Anreicherung der gewählten Zielregionen infrage kommen. Für die Auswertung der NGS-Daten stehen zahlreiche bioinformatische Programme zur Verfügung, die allerdings durch in der molekularen Diagnostik sehr erfahrene Wissenschaftler/Ärzte verwendet werden sollten.

Im Rahmen der BEACON-CRC-Studie, die in insgesamt 221 Zentren in 28 Ländern durchgeführt wurde (davon 111 Zentren in Europa), ergab die Auswertung der für die BRAF-Bestimmung von 510 Proben eingesetzten Verfahren folgendes Bild: In 48,8 % der Untersuchungen wurden für die BRAF-Testung noch immer Einzelgennachweise geführt. Proteinbasierte Verfahren (Immunhistologie) kamen in 0,7 % der Analysen zum Einsatz. Die Mehrheit der BRAF-Testungen wurde allerdings zusammen mit dem Nachweis weiterer Genveränderungen (z. B. als fokussierte, amplikonbasierte NGS) durchgeführt (50,5 %). Die zwischen lokaler und zentraler Testung beobachtete Abweichung zeigt dabei die Relevanz einer Standardisierung von diagnostischen Verfahren, insbesondere im Hinblick auf die zunehmende Bedeutung zielgerichteter Therapieansätze. Eine eindeutige Bestätigung der lokal nachgewiesenen *BRAF*^V600E^-Mutation erfolgte lediglich in 90,7 %. Diese Diskrepanz basierte bemerkenswerterweise überwiegend auf einer ungenügenden Anzahl Tumorzellen in den Proben – aller Wahrscheinlichkeit nach aufgrund der Tatsache, dass die BRAF-mutierten Tumore grundsätzlich mit einem muzinösen Adenokarzinom assoziiert sind, welches weniger Tumorzellen enthält. Bei 1,6 % der zentralen Wiederholungstests wurde das lokale Ergebnis eindeutig negiert. Diese Möglichkeit einer Diskonkordanz zwischen lokaler und zentraler Testung in Betracht ziehend erlaubte das Studienprotokoll zwar den Einschluss von Patienten basierend auf einem lokalen *BRAF*^V600E^-Mutationsnachweis im Rahmen des molekularen Prescreenings, forderte als Einschlusskriterium aber zudem und binnen 30 Tagen nach erstmaligem Erhalt der Studienmedikation die zentrale Bestätigung. Nachdem die Studie die präspezifizierte Anzahl abweichender Testergebnisse erreicht hatte, wurde das Vorliegen des zentralen Nachweises durch den in den USA als Companion Diagnostic entwickelten Assay zur Voraussetzung für die Aufnahme aller weiterer Patienten.

Zwischen der Europäischen Union (EU) und den USA bestehen Unterschiede in der Regulierung von In-vitro-Diagnostika (IVD). Solche Tests unterliegen in den USA einer zentralen Zulassung (Premarket Approval) durch die Food and Drug Administration (FDA), während in der EU eine dezentrale Zulassungsphilosophie vorherrscht, bei der die Hersteller die Konformität ihres Tests durch eine „Benannte Stelle“ („notified body“) bewerten lassen können und danach die sog. CE-Kennzeichnung („Conformité Européene“) selbst auf dem Produkt bzw. Test aufbringen^2^. Des Weiteren erteilt die FDA Zulassungen für Arzneimittel bei den zielgerichteten Therapien häufig nur in Verbindung mit einem festgelegten und zeitgleich ebenfalls zugelassenen Companion Diagnostic, das zum Zulassungsbeginn häufig marktexklusiv für das entsprechende Arzneimittel ist. Die Verwendung des Companion Diagnostic ist somit Voraussetzung zur Verschreibung des Arzneimittels durch den Arzt [54].

Die US-amerikanische Gesetzgebung unterscheidet solche standardisierten, zumeist kommerziell erhältlichen Tests von den „laboratory-developed tests“ (LDT), die – siehe die klassischen Testverfahren – von Instituten in Eigennutzung konzipiert, validiert und angewendet werden. Seitens der Deutschen Akkreditierungsstelle (DAkkS) werden die LDT auch als In-house-Tests bezeichnet. Diese unterliegen in der Regel keiner Zulassung oder Kennzeichnungspflicht. Es gibt allerdings einen Leitfaden der DAkkS zur Validierung molekularpathologischer Untersuchungsverfahren [55, 56]. In den USA gibt es seit Januar 2017 ein Positionspapier, in dem eine stärkere, prospektive Regulierung von LDT aufgrund der zunehmenden prognostischen und prädiktiven Bedeutung propagiert wird – dies gilt insbesondere auch für die Stellung und stetig wachsende Bedeutung des NGS [57, 58]. Mit dem FoundationOne® CDx (F1CDx) Test hat Ende 2017 erstmalig ein NGS-Verfahren in den USA eine Zulassung als Companion Diagnostic erhalten (Tab. 3; [59]).

Vor diesem Hintergrund lässt sich die Vielfalt der konkurrierenden klassischen und neuen DNA-analytischen Verfahren zur *BRAF*-Mutationsbestimmung leichter einordnen. Von den kommerziellen, allelspezifische PCR-Techniken einsetzenden Verfahren ist in den USA derzeitig einzig der Qiagen *therascreen*® Test beim *BRAF*-mutierten mKRK empfohlen, in Europa liegt eine CE-Kennzeichnung vor. Es ist zu erwarten, dass es auch für die derzeitig nur beim Melanom zugelassenen Tests bald entsprechende Anpassungen in den USA geben wird, das KRK betreffend. In der BEACON-CRC-Zulassungsstudie waren als Verfahren ausschließlich die PCR und NGS basierend auf lokalen Assays im Studienprotokoll erlaubt [44].

Ein Vergleich zwischen kommerziellen (d. h. FDA-zugelassenen) Tests und LDT führte für die *EGFR*-, *KRAS*- und *BRAF*-Testung zum Ergebnis, dass es in der Assayperformance keinen Gesamtunterschied zwischen den Methoden bezüglich der 3 untersuchten Gene gibt, die durchschnittliche Analysegenauigkeit betrug 97 % [60]. Vor dem Hintergrund der zuvor für das KRK dargestellten Notwendigkeit zur Testung von *KRAS, BRAF, MSI‑H*/*dMMR, MLH1* und ggf. weiteren Genen steht in der pathologischen Praxis heute natürlich die Paneldiagnostik im Fokus – in Deutschland setzen alle universitären und große nichtuniversitären Einrichtungen dafür mittlerweile fokussiertes NGS ein. Hierbei kommen als Plattformen häufig die Systeme von Illumina (MiSeq™ oder NextSeq™) und Thermo Fisher (*Ion Gene Studio S5*™) zum Einsatz [61–63]. Im Rahmen einer deutschlandweiten multizentrische Validierungsstudie konnte eine hohe Überstimmung zwischen verschiedenen NGS-Plattformen und Genpanels gezeigt werden. Getestet wurden neben dem KRK auch Proben von Lungen- und Mammakarzinomen [61].

### Immunhistochemische Verfahren

Neben den DNA-analytischen Verfahren stellen proteinbasierte Analysen unter Verwendung des VE1-Antikörpers eine Alternative dar zur molekularpathologischen Testung von *BRAF*^*V600E*^. Letztere gilt weithin als der Goldstandard bei der *BRAF-*Mutationstestung [50, 64]. Zugleich aber ist der proteinbasierte immunhistologische Nachweis, der auch bei der MSI-Testung Einsatz finden kann, die einzige sinnvoll praktizierbare Methode zur Bestimmung der Expression mutanten *BRAF*-Proteins. Die Spezifität des Verfahrens liegt bei 98–100 %, die Sensitivität bei 85–100 % [49, 50], die In-Lab-Turnaround-Zeit einen Tag. Die Verlässlichkeit der Methode ist somit grundlegend gegeben, allerdings bleiben einige Herausforderungen, wie zum Beispiel die Etablierung eines verlässlichen Scorings der Proteinexpression [64]. Die Bestimmung der Immunfärbung zur Bestimmung *BRAF*-mutierten Proteins bietet sich somit zwar als schneller und kostengünstiger Test an, jedoch ist beim mKRK mittlerweile die Bestimmung multipler Alterationen erforderlich, sodass DNA-analytische Verfahren heutzutage sicherlich bevorzugt zur Anwendung kommen sollten.

Chu et al. untersuchten populationsbasiert die Ergebnisse der immunhistochemischen Testung (IHC) und NGS-Testung (BRAF^V600E^) [65]. Die Rate falsch positiver IHC-Tests betrug 17 %, wobei nur 43 % aller IHC-getesteten Patienten per NGS überprüft wurden. NGS-getestete Patienten hatten ein besseres medianes OS bei jüngerem Alter, geringerer Rate synchroner Metastasen und höherer Therapierate. Die Autoren kamen zu dem Schluss, dass NGS zwar die Standardtestung sein sollte, sollte NGS jedoch nicht unmittelbar zeitnah zugänglich sein, stellt die IHC einen sinnvollen Screeningtest mit rascher Ergebnisverfügbarkeit dar, zumal mittels reflexiver IHC-Testung 57 % mehr *BRAF*-mutierte mKRK identifiziert werden konnten als durch Standard-NGS-Einsatz.

## Fazit für die Praxis

- Der Nachweis einer *BRAF*^*V600E*^-Mutation ist insbesondere vor dem Hintergrund einer Mikrosatellitenstabilität (MSS) mit einer sehr schlechten Prognose assoziiert und stellt somit einen aggressiven Subtyp des kolorektalen Karzinoms (KRK) dar.
- Patienten mit *BRAF*^*V600E*^-mutiertem metastasiertem KRK (mKRK) weisen somit einen hohen „unmet medical need“ auf. Mit Encorafenib + Cetuximab steht nunmehr ein chemotherapiefreier, zielgerichteter Therapiestandard in der Zweit- und Drittlinie zur Verfügung. Die Aufnahme der kombinierten BRAF- und EGFR-Blockade in die deutschen/europäischen Behandlungsalgorithmen und Guidelines ist in den kommenden Monaten zu erwarten.
- Im Sinne einer adäquaten Planung der Therapiesequenz ist daher bei allen Patienten mit einem mKRK vor Einleitung der Erstlinientherapie eine *BRAF*-Testung unbedingt erforderlich.
- Für die Testung stehen eine Vielzahl von Methoden zur Verfügung, wobei die Paneldiagnostik mit Next Generation Sequencing – insbesondere in Kombination mit dem Nachweis von weiteren molekularen Alterationen – bevorzugt zur Anwendung kommen sollte.

## Footnotes

1 Bezüglich der Leitlinien der European Society of Medical Oncology (ESMO) gibt es neben den seit 2016 zuletzt neu publizierten „*Consensus Guidelines*“ für das metastasierte KRK (siehe Ref. [4]) die Anfang 2018 publizierten „*Pan-Asian Adapted ESMO Consensus Guidelines*“. Auch wenn in diesem Artikel auf die etwas ältere ESMO Guideline referenziert wird, gilt der Bezug immer für beide Guidelines und die darin abgebildete aktuelle Studienevidenz.

2 Mit der **CE-Kennzeichnung **erklärt der Hersteller, Inverkehrbringer oder EU-Bevollmächtigte gemäß EU-Verordnung 765/2008, „*dass das Produkt den geltenden Anforderungen genügt, die in den Harmonisierungsrechtsvorschriften der Gemeinschaft über ihre Anbringung festgelegt sind*“.
